# Cardiovascular diseases and vulnerable plaques: data, modeling, predictions and clinical applications

**DOI:** 10.1186/1475-925X-14-S1-S1

**Published:** 2015-01-09

**Authors:** Dalin Tang, Zhi-Yong Li, Frank Gijsen, Don P Giddens

**Affiliations:** 1 School of Biological Science and Medical Engineering, Southeast University, Nanjing 210096, China; 2 Department of Mathematical Sciences, Worcester Polytechnic Institute, Worcester, MA 01609, USA; 3 Department of Biomedical Engineering, Thorax center, ErasmusMC, Rotterdam, The Netherlands; 4 Wallace H. Coulter Department of Biomedical Engineering, Georgia Institute of Technology and Emory University, Atlanta, GA, 30332, USA

## Introduction: two symposia on "cardiovascular diseases and vulnerable plaques"

Cardiovascular disease (CVD) is the leading cause of death worldwide. Huge effort has been made in many disciplines including medical imaging, computational modeling, biomechanics, bioengineering, medical devices, animal and clinical studies, population studies as well as genomic, molecular, cellular and organ-level studies seeking improved methods for early detection, diagnosis, prevention and treatment of these diseases [1-14]. However, the mechanisms governing the initiation, progression and the occurrence of final acute clinical CVD events are still poorly understood. A large number of victims of these diseases who are apparently healthy die suddenly without prior symptoms. Available screening and diagnostic methods are insufficient to identify the victims before the event occurs [8,9]. Most cardiovascular diseases are associated with vulnerable plaques. A grand challenge here is to develop new imaging techniques, predictive methods and patient screening tools to identify vulnerable plaques and patients who are more vulnerable to plaque rupture and associated clinical events such as stroke and heart attack, and recommend proper treatment plans to prevent those clinical events from happening.

Articles in this special issue came from two symposia held recently focusing on "Cardiovascular Diseases and Vulnerable Plaques: Data, Modeling, Predictions and Clinical Applications." One was held at Worcester Polytechnic Institute (WPI), Worcester, MA, USA, July 13-14, 2014, right after the 7^th ^World Congress of Biomechanics. This symposium was endorsed by the World Council of Biomechanics, and partially supported by a grant from NIH-National Institute of Biomedical Image and Bioengineering. The other was held at Southeast University (SEU), Nanjing, China, April 18-20, 2014.

## Invited speakers, presenters, and participants

The objective of the two symposia was to invite experts and researchers world-wide in the cardiovascular diseases and vulnerable plaque research area to exchange the most updated research techniques and findings, to provide training to newer researchers and students, and form a joined force to push the image-based research effort closer to clinical applications. Invited speakers and participants at the WPI symposium included Don Giddens and David Molony (Georgia Tech), Jonathon Gillard and Zhongzhao Teng (Cambridge University, UK), Peter Stone (Harvard Medical School), David Saloner (UCSF), Jolanda Wentzel and Frank Gijsen (Erasmus Medical Center, the Netherlands), Natalia Maldonado from Sheldon Weinbaum's group (CUNY), Rupak Banerjee and Namheon Lee (University of Cincinnati), Kristen Billiar, Glenn Gaudette, Mayer Humi, Roger Lui, Marcus Sarkis, and Zheyang Wu (WPI), Umberto Morbiducci and Diego Gallo (Politecnico di Torino, Italy), Michael Walsh, Hilary E. Barrett and Cunnane Eoghan (University of Limerick, Limerick, Ireland), Shunichi Kobayashi (Shinshu Univ, Japan), Haichao Han (University of Texas at San Antonio), Susan Lessner (University of South Carolina School of Medicine), Takeo Matsumoto and Shukei Sugita (Nagoya Institute of Technology, Japan), Katherine Zhang (Boston University), Liang Wang and Heng Zuo (WPI), and many others. Abstracts presented at the WPI symposium provided here for easy reference [32-49].

Invited international and domestic speakers and participants at the SEU symposium included Zahi Fayad (Mount Sinai), David Saloner (University of California San Francisco), Danny Bluestein (Stony Brook University), Frank Gijsen (Erasmus Medical Center), Rita Z. Goldstein (Icahn School of Medicine at Mount Sinai), Shunichi Kobayashi (Shinshu University), Quan Long (Brunel University, UK), Yubo Fan (Beihang University), Mian Long (Chinese Academy of Sciences), Jiang, Zonglai Jiang (Shanghai Jiaotong University), Ning Gu (Southeast University), Naifeng Liu (Zhongda Hospital, Southeast University), Gaojun Teng (Zhongda Medical School, Southeast University), Zhi-Yong Li (Southeast University), Keqiang Wang (Medical School, Fudan University), Weiyi Chen (Taiyuan University of Science and Technology), Xiaoyan Deng (Beihang University), Fabao Gao (Sichuan university), Xueying Huang (Xiamen University), Liang Li (Sichuan University), Youjun Liu (Beijing University of Technology), Ai'ke Qiao (Beijing University of Technology), Shengzhang Wang (Fudan University), Li Yang (Chongqing University), Yuyu Yao (Zhongda Hospital, Southeast University), Qi Yuan (Xi'an Jiaotong University), Wen Zeng (Sichuan Primed Bio-tech Co., Chengdu, China), Yiyi Zhan (South China University of Technology), Xizheng Zhang (Academy of Military Medical Science), Jian Zhu (Zhongda Hospital, Southeast University), and many others. Abstracts presented at the SEU symposium provided here for easy reference [50-74].

## Specific themes of the symposia

Due to the complexity and multi-disciplinary nature of the problems faced by the researchers, we need to combine medical imaging and modeling with other modalities for better potential in patient screening and clinical event prediction. Computational modeling should be integrated with in vivo intravascular ultrasound (IVUS), angiography, Magnetic Resonance Imaging (MRI), mechanical testing, and histological analysis to analyze vulnerable atherosclerotic plaques and identify critical blood flow and plaque stress/strain indicators which may be used for quantitative carotid/coronary plaque vulnerability assessment. The papers presented with intensive panel discussions at the two symposia were focused on the following four specific themes:

Theme 1. Identify critical issues encountered in medical image acquisition related to cardiovascular diseases and vulnerable plaque research and develop corresponding strategies including image resolution, disease and vulnerable plaque identification, cap thickness, intraplaque hemorrhage, and thrombosis;

Theme 2. Identify critical issues in mechanical testing and quantification of material properties and other boundary conditions such as vessel branching, flow rate and velocity specifications, surface inflammation and blood pressure;

Theme 3. Identify critical issues in model development, disease and plaque assessment, mechanisms, prediction of rupture and clinical events; discuss issues related to method and model sharing and training of students and junior researchers. Conduct Image-Based FSI Modeling Workshop;

Theme 4. Validate model predictions and assessment plan. Discuss how clinicians can use mechanical and modeling analysis in clinical and surgical applications; Medical device (stent) improvement; Research dissemination and commercialization, transforming research to clinical practices.

## Problems and topics discussed

The invited speakers and participants at the symposia had intensive discussions on the following critical issues and major challenges:

a) Gold standard for in vivo assessment of vulnerable plaques. Histology has been serving as the gold standard for imaging technique development, as well as plaque assessment and classifications. However, such a gold standard does not exist for investigations based on in vivo data. How do we proceed to establish such gold standards and bench marks for plaque assessment?

b) It is known that MRI and IVUS still need to have better resolution to better quantify thin plaque caps, lumen surface conditions, plaque components, and detect vulnerable plaques. What are the possible ways to further develop imaging techniques to meet those needs?

c) How could we use in vivo imaging techniques to determine blood pressure and material properties? What are the barriers and strategies to overcome them?

d) What are the basic skills needed in constructing FSI models based on in vivo data? What are the strategies to reduce the intensive model construction labor? Best practice for sharing?

e) What are the challenges in discovering possible mechanisms governing plaque progression and rupture? How could we validate our findings?

f) How do we identify potential risk factors and quantify their prediction power for clinical events?

g) Strategies to bring research closer to realistic patient screening and diagnostic applications.

## Future directions

Discussion of the topics leads to future tasks and directions. Interdisciplinary collaborations will definitely be needed. Researchers should go beyond their own expertise to understand the bigger picture so that they could further advance their own area.

a) Medical images need to improve resolution so that models can have more accurate data;

b) Image-based models will include more complete in vivo data and make better predictions;

c) Predictive methods should be developed and validated by clinical follow-up studies for potential clinical implementations;

d) Data and model sharing will help to advance research closer to "precision medicine";

e) Bring research to practice: software development, medical devices, and commercialization.

## Papers selected in this issue

Papers selected in this special issue cover a variety of new areas and topics. New modeling papers including work by Molony et al. showing significant differences for models with and without coronary branches [15], Lu et al. paper for neovessels with intraplaque hemorrhage [16], Sanyal and Han's paper for plaques with buckling [17], Barrett et al.'s paper characterizing human atherosclerotic carotid plaque tissue composition and morphology using combined spectroscopic and imaging modalities [18], and Liu's paper investigating influence of model boundary conditions on blood flow patterns in coronary plaque models [19]. Cunnane et al. used simulation of human atherosclerotic femoral plaque tissue to study the influence of plaque material model on numerical results [20]. Ventricle model papers included a review paper by Lee et al. [21] and a mechanical analysis paper by Gan et al. [22]. Experimental and numerical studies of the circle of Willis were presented by Zhu et al. [23] and Ren et al. [24]. Several papers presented new imaging techniques for better resolutions and/or applications [25-28]. Papers by Chen et al. and Liu et al. represented some effort in medical device and clinical interventions [29,30]. Das' et al. paper represented an effort in automating numerical method for potential software implementation and commercialization [31], even though some simple samples were used for illustration. This is certainly a limited collection of papers from the participants of the symposia. Rather than presenting results and providing solutions, the purpose of the symposia was to identify problems and future directions. Some of those were given in Sections 4 & 5. We need all the wisdom we can get collectively from all the disciplines and researchers to overcome the challenges we are facing.

## Competing interests

Other than the grants listed in the acknowledgement section, the authors declare that they have no other competing interest.

## Authors' contributions

All authors actively contributed to the research and the writing of the manuscript. DT and ZYL were guest editors for the special issue. DT and ZYL were the organizers for the SEU symposium. DT, FG and DPG were the organizers for the WPI symposium. Professors David N Ku and Danny Bluestein were organizers for the WPI symposium but could not attend due to last-minute schedule conflict. All authors have made substantial contributions and have been involved in drafting the manuscript or revising it critically for important intellectual content; and have given final approval of the version to be published. Each author has participated sufficiently in the work to take public responsibility for appropriate portions of the content.

## Declarations

Publication of this supplement has not been supported by any sponsorship. Publication of this article was paid with funding from Tang's WPI funds. Information about the source of funding for other articles can be found in the individual articles.

This article has been published as part of *BioMedical Engineering OnLine *Volume 14 Supplement 1, 2015: Cardiovascular Disease and Vulnerable Plaque Biomechanics. The full contents of the supplement are available online at http://www.biomedical-engineering-online.com/supplements/14/S1
